# E-Leadership and Teleworking in Times of COVID-19 and Beyond: What We Know and Where Do We Go

**DOI:** 10.3389/fpsyg.2020.590271

**Published:** 2020-12-11

**Authors:** Francoise Contreras, Elif Baykal, Ghulam Abid

**Affiliations:** ^1^School of Management and Business, Universidad del Rosario, Bogotá, Colombia; ^2^School of Business and Management Sciences, Istanbul Medipol University, Istanbul, Turkey; ^3^Department of Business Studies, Kinnaird College for Women, Lahore, Pakistan

**Keywords:** e-leadership, teleworking, COVID-19, virtual teams, remote work environments

## Abstract

Suddenly, COVID-19 has changed the world and the way people work. Companies had to accelerate something they knew was imminent in the future, but not immediate and extremely humongous. This situation poses a huge challenge for companies to survive and thrive in this complex business environment and for employees, who must adapt to this new way of working. An effective e-leadership, which promotes companies’ adaptability, is needed. This study investigates the existing knowledge on teleworking and e-leadership; and analyzes the supposed challenges. The literature review shows that companies with effective e-leadership can view teleworking as an opportunity. It is advantageous for not only companies’ productivity but also the environment and people who work remotely. However, a traditional or no leadership can result in some risks. Thriving in remote work environments implies that managers must adjust the companies’ structure, making them less hierarchical, and developing new abilities to establish a strong and trustworthy relationship with their employees to maintain their competitiveness, while retaining a genuine concern for their employees’ well-being. Similarly, successful e-leadership must be able to consolidate and lead effective virtual teams to accomplish organizational goals. This study contributes to the literature and leaders during the pandemic.

## Introduction

In the past few months, telework or working from home has experienced rapid growth owing to the pandemic, leading to significant changes in work methods. İt refers to a flexible working method that is not limited by time, location, type of communication technology, and the use of information. The successful implementation of this requires technology, social, and organizational support specifically in the form of e-leadership practices where the emergence of digital technology and Internet services has facilitated the progress of teleworking. The current pandemic (COVID-19) has generated a massive and sudden change in how companies operate. After the outbreak of COVID-19, social distancing, which means a deliberate physical space between individuals, has been adopted as a sound prevention method (Prin and Bartels, 2020) and thus necessitated remote working. In this context, information and communication technologies (ICTs) allow employees to work anytime and almost everywhere (Müller and Niessen, 2019). Moreover, teleworking was imminent, but the pandemic has made it a compulsion. It is speculated that this new global work norm would continue even after the pandemic is overcome. This change has deeply impacted not only how organizations operate but also the relationship between employees and employers. Thus, in this new work environment with possible risks (see Bouziri et al., 2020; Lambert et al., 2020), opportunities, and flexible work arrangements, leadership practices cannot be the same. Leadership practices must adapt to new remote or virtual conditions for effective leadership and sustainable performance. This is why Bennis (2009) on his famous book “on becoming a leader” argued that leaders are not born they are made. Leaders should transform themselves to achieve organizational goals by engaging teleworkers who enjoy a fruitful virtual work environment and allow them to thrive in their work. Undoubtedly, leadership in this new labor reality will be decisive for organizations to survive and grow. As nature has demonstrated and this can be applied to companies, if companies do not respond to crises and adapt to the new conditions, they are likely to disappear. Based on a literature review (from 2000 to 2020), this study investigates the existing knowledge about teleworking and e-leadership and pre- and post-COVID-19 risks and opportunities for organizations. Between March and July 2020, we carried out this literature review, looking for scientific publications on telework and e-Leadership in academic journals-databases (Web of Sciences, PsychINFO, SCOPUS, SciELO). The literature search was carried out using the following keywords and combinations between them: Telework, e-leadership, telecommuting and e-leadership, virtual environments, virtual work, virtual teams, telework and COVID-19. Non-recent articles were excluded unless they were quite relevant. The body of the retrieved literature was reviewed and organized for presentation in this document. From more than one hundred articles, we identified and synthesized the findings and contributions of about 80 academic publications, specifically peer-reviewed articles.

The present study revolves around understanding the association between teleworking, leadership and e-leadership that represents the emergence of leadership in the e-environment context where the work is mediated by information technologies, high complexity and a changing working environment that makes imperative for leaders to change their practices, attitude, and behavior for long term organizational sustainability. In order to better comprehend the above phenomena, this study is structured as follows. In section “Teleworking and the Emergence of COVID-19,” we discuss the opportunities and risks with teleworking with the emergence of COVID-19. Section “Management, Leadership and Telework Environments” deals with understanding the management and leadership in the environment of teleworking. In section “E-leadership and its Conceptualization,” we discuss the phenomenon of e-leadership and its conceptualization. In section “E-leadership, Teleworking and Virtual Teams,” the association among e-leadership, teleworking and virtual teams is analyzed. Finally, in section “Conclusion and Propositions for Further Studies,” we put forth some propositions for further studies.

## Teleworking and the Emergence of COVID-19

In the past decades, companies have evolved according to new conditions of the work environment, such as globalization, fierce competition, new demographic structures, and increasing development of ICTs (Wojcak et al., 2016). The transition from the industrial era to a digitalized business environment led to a shift from a mechanistic perspective to a more organic perspective, where organizations embrace flexible structures (Pulley and Sessa, 2001). After 2000s, work has been increasingly detached from on-site (Felstead and Henseke, 2017) to facilitate the workforce and to provide better services to the customers. Therefore, teleworking was steadily growing globally in several sectors. Among these sectors, service industry encompasses the highest overall percentage of workforce who work remotely (17%), followed by health care industry (12%), finances and insurance industry (10%), manufacturing sector (8.5%), and education industry (7.5%) (He et al., 2020). Teleworking is always debated because of the blurring boundaries regarding non-work and work, personal, and social effects of not being physically present at a job, and the risks and benefits of flexible working hours. Under traditional conditions (e.g., before COVID-19), teleworking was needed temporarily (Allen et al., 2015). However, in this current pandemic situation, most of the employees around the world are full time away from the office and working from home. Thus, this pandemic has suddenly changed how people work and it is not yet very clear how long we have to continue working from home in different countries.

The World Health Organization (WHO) officially announced the outbreak of coronavirus disease on March 11, 2020, as a pandemic and suggested preventive measures to contain its spread. Telework was an important measure suggested by World Health Organization (2020) and successfully implemented by organizations and governments around the world. Thus, since March 2020, more than 3.5 billion individuals have been confined to their homes, which meant that several millions were teleworking (Bouziri et al., 2020). This teleworking may lead to social or professional isolation, which is referred to as the missing of the everyday social aspect of work because employees are physically away from other workmates, hence leading to not being actively participating in information sharing and co-learning. This feeling of professional isolation adversely affects job performance (Golden et al., 2008) because employees do not have their supervisor and colleagues’ support in problem solving as they would if they were physically present at work. In this context, the role of e-leadership lies in facilitating the work conditions and keeping employees motivated toward achieving the desired goals. This situation calls for a different type of leadership, known as e-leadership, which entails the development of distinct abilities to improve organizational functioning in virtual and remote work environments (Roman et al., 2019).

Before the COVID-19, teleworking was steadily growing globally across many sectors. The pandemic accelerated this process and now companies must operate with employees having to work in places different from the traditional workplace through teleworking. In fact, teleworking was popular even before the pandemic (He et al., 2020) and the infrastructure for teleworking already existed. Hence, the adoption of this working style has been relatively easy for several companies (Béland et al., 2020). Tietze and Musson (2005) asserted that the future of work will be “flexible, mobile, temporary and mediated by technology” (p. 1331), that is, by teleworking. Telework, telecommuting, or working remotely is a wide-ranging concept that covers any paid work performed from a distance in any place different from the physical presence in the organization where employees meet organizational objectives through ICTs, sometimes managing their own time under less direct supervision (Wojcak et al., 2016). These employees usually work remotely with autonomy for at least a few days of their labor time (Nayani et al., 2018). However, Bentley (2014) highlighted the importance of delimiting the notion of telework to avoid confusion with employees who work for companies from outside, such as those who work in call centers or as freelance employees.

### Opportunities of Teleworking

Teleworking has some potential advantages. Empirical studies have found favorable outcomes of teleworking such as job performance, job satisfaction, lesser work-family imbalance, reduced rates of stress, and lesser turnover intentions (Kossek et al., 2006; Fonner and Roloff, 2010; Coenen and Kok, 2014; Vega et al., 2015). Likewise, Othman et al. (2009) demonstrated the positive effect of teleworking on employees’ work-life balance. Additionally, Azarbouyeh and Naini (2014) stated that teleworking is effective in enhancing the quality of life, whereas, Kazekami (2020) found that teleworking improves employees’ happiness and work satisfaction. However, the benefits are evident where the employees find managerial, peer, and technological support. This support helps reduce any potential negative impacts arising from social isolation, mitigate the work-family conflict, and reduce the stress (Bentley, 2014).

Teleworking can also influence on the reputation and corporate image because green companies are concerned about the environment. Currently, heavy traffic and air contamination are some of the most relevant global issues (Giovanis, 2018). Teleworking is a viable short and long-term solution to improve the quality of air mainly in urban areas while improving the quality of life (Giovanis, 2018). Consequently, the world will witness less contamination because employees do not have to use daily transport, thus saving time and money. Interestingly, the term “telecommuting” was used for the first time in the 1970s to relieve traffic and reduce pollution through flexible and better work-life balance (Nilles, 1998). Another advantage of a highly complex work environment is that companies have access to specialized expertise, regardless of the team members’ location, which allows companies to find more creative solutions to this complex global work environment (Malhotra et al., 2007). Similarly, digitalization, new communication tools, and more availability and speed of information increase the efficiency and process of standardization (Cortellazzo et al., 2019). For employees, teleworking offers more flexibility to deal with family matters because they can work anywhere and anytime, thus improving the family atmosphere (Fedakova and Ištoňová, 2017), and the autonomy to manage time allows them to harmonize their personal and work duties (Wojcak et al., 2016). Hence, it increases job opportunities for women and employees with disability (Morgan, 2004).

Furthermore, work autonomy through free choice to directly influence one’s working time, place, and methods is associated with higher productivity (Pavlova, 2019). Moreover, in their meta-analysis of 46 studies Gajendran and Harrison (2007) showed that telecommuting lowers turnover intentions and stress. The absence of an immediate supervisor and a less formal working atmosphere reduces the work stress for employees. Moreover, teleworking helps employees create their own rhythm of work and prevents distractions from other employees (Kłopotek, 2017). Additionally, it decreases the individual and organizational burdens of absenteeism because it allows employees to fulfill their work obligations even in times when there is trouble reaching the office, allowing employees to fulfill their duties (Nakrošienė et al., 2019). Indeed, these advantages contribute to greater organizational commitment, job satisfaction, and well-being.

### Risks of Teleworking

Some risks posed by teleworking must be considered, namely, social isolation from work teams (Pyoria, 2011). Social isolation leads to employees being disconnected from the working environment leading to lower performance and gradual demotivation (Wojcak et al., 2016; Fedakova and Ištoňová, 2017). Long-term isolation has adverse effects on employees’ performance and increases turnover intention, family-work and work-family conflict (Golden et al., 2008). In work-to-family conflict individuals are hindered to meet role demands in their private life because of work demands while in the family-to-work conflict, they can be hindered to meet their private roles because of home demands. Their study also empirically revealed that volition, perceived work pressure and perceived home pressure are all relevant for understanding employees’ work-to-home conflict rather than home-to-work conflict and work-home practices to be beneficial employees should not feel pressure to either use or not use offered practices (Delanoeije and Verbruggen, 2019). Furthermore, as Cooper and Kurland (2002) indicate teleworking reduces the learning benefits that people enjoy when working in the same workplace. Moreover, teleworking requires greater organizational skills (Kłopotek, 2017); it is suitable for only self-organized people who are successful in time allocation. On the one hand, teleworking can lead to anxiety among employees about the possible shrinking of career prospects owing to reduced visibility (Maruyama and Tietze, 2012), and unfortunately the advantages of teleworking come at the cost of intensified work. Therefore, a commonly cited concern of managers regarding teleworking is the possibility of decreased job performance. In other words, the lack of trust in employees’ ability and willingness to perform at the same level compared with what they could attain if they were to work with their manager in the same place (Kaplan et al., 2018). Digital environments have some common problems, such as email/data overload, employees’ alienation, weak social relationships, poor accountability in teams, low trust, insufficient technological skills, and an inability to influence change based on commitment (Van Wart et al., 2019).

Finally, telework raise ethical concerns for e-leaders, such as exploitation of employees with work and information overload that overlap with domestic and work settings, resulting in an intrusion into employees’ personal life (Cortellazzo et al., 2019; Gálvez et al., 2020). Although teleworking gives individuals greater autonomy in terms of time and space, the simultaneous use of different normative control mechanisms under the guise of autonomy leads to work intensification and extra burden to employees. This obscure control mechanism results in greater self-regulation and promotes greater work efforts from employees (Bathini and Kandathil, 2019). Moreover, individuals who are grateful for the flexibility provided by teleworking make greater effort and achieve higher performance, ending up with a higher sacrifice than with traditional working methods (Putnam et al., 2014). Table 1 shows the main reported findings of opportunities and risks of teleworking.

**TABLE 1:** Opportunities and risks of teleworking.

Opportunities	Source	Risks	Sources
Offers job opportunities for people with disabilities and for women increases job opportunities for women and employees with disability.	Morgan, 2004	Reduction of the learning benefits that is available when people are working in the same workplace.	Cooper and Kurland, 2002
Global workforce available, access to a specialized knowledge regardless of geographic location.	Malhotra et al., 2007	Social and professional isolation.	Cooper and Kurland, 2002; Golden et al., 2008; Pyoria, 2011; Bentley, 2014
Greater competitiveness to successfully insert in global work environments.	Avolio et al., 2014; Narayanan et al., 2017	Employees concerns due to the reduction of career prospects by feeling less visible.	Maruyama and Tietze, 2012
Lower stress, lesser turnover intentions, lesser work-family imbalance and job satisfaction.	Kossek et al., 2006; Gajendran and Harrison, 2007; Fonner and Roloff, 2010; Coenen and Kok, 2014; Vega et al., 2015	Because the flexibility, highly motivated employees can work more hours than in traditional work environment, resulting in exhaustion.	Putnam et al., 2014
Autonomy and flexibility at work allow harmonizing the personal and work matters favoring the workers’ well-being.	Fedakova and Ištoňová, 2017	Physical distance and cultural diversity threaten trust building, commitment and cohesion among the team members.	Hoch and Kozlowski, 2014
Information availability increases job performance.	Schwarzmüller et al., 2018	Lower job performance and demotivation.	Golden et al., 2008; Wojcak et al., 2016; Fedakova and Ištoňová, 2017
Contribute to the solution of global problems such as pollution and air quality, while influencing the firms’ reputation.	Giovanis, 2018	Work-home conflicts.	Golden et al., 2008; Bentley, 2014; Delanoeije and Verbruggen, 2019
The team members’ heterogeneity promotes creativity and innovation through a combination of various perspectives to achieve an objective.	Gupta and Pathak, 2018	Work and information overload that overlap with domestic and work settings.	Cortellazzo et al., 2019; Gálvez et al., 2020
Decreases absenteeism due to employees do not have to face difficulties to reach the workplace.	Nakrošienė et al., 2019		
Opportunity to interact and establish effective virtual teams, increasing their creative capacity.	Malhotra et al., 2007; Schwarzmüller et al., 2018; Cortellazzo et al., 2019		
Work autonomy and less distraction potentially allow higher productivity.	Kłopotek, 2017; Pavlova, 2019		

*Source: Authors own elaboration.*

## Management, Leadership and Telework Environments

Leadership has several definitions; however, generally leadership can be defined as an influence process to achieve organizational goals. In the traditional work environment, this influence is exerted by not only formal leaders but also employees without formal authority (informal leadership). In teleworking, the influence of formal leaders is more obvious. They must influence to build effective and functional virtual teams to reach organizational goals. Before analyzing the concept of leadership in virtual environments, this study makes the following propositions supported in the literature on leadership: (1) there is no leader without followers; (2) one can be considered a leader only when people recognize him or her as such; (3) leadership can be considered an interactive process of social influence and it is based on relationships; and (4) as a result of effective leadership, employees make their best effort to accomplish organizational goals. Hence, in addition to the formal authority, leaders must develop the ability to influence others to get work done.

Beyond the polemic and the unfinished debate about whether leadership and management should be conceived as the same construct (Mintzberg, 2009) or distinct (Kotterman, 2006), in teleworking the role between one and the other appears more distinct than in traditional workplaces. Teleworking brings more challenges for leaders than managers. In other words, teleworking is more feasible and even improves the efficiency of the traditional role of management (i.e., planning, budgeting, control and establishing administrative procedures) than exerting effective leadership (i.e., influence others to achieve organizational goals) through electronic devices. According to Nayani et al. (2018), both leadership and management are equally important in teleworking. However, adapting traditional leadership practices to a technologically mediated environment is more complicated (Pulley and Sessa, 2001). A distributed workforce must be led by adopting new and more complex methods in communication, performance management, training, and relationship building (Flood, 2019).

From the management perspective, teleworking can be favored by flatter and more decentralized structures (Cortellazzo et al., 2019). The increase in connectivity within the companies in addition to information availability contributes to diminishing hierarchies and organizational boundaries, leading to companies working by projects more than traditional activities and thus, employees participate in the creation of value for the companies (Cortellazzo et al., 2019). Owing to information availability, the power of the company tends to be more distributed and less centralized, involving employees in the decision-making process. This participative decision-making helps leaders analyze and prioritize relevant information from the large amount of available data, respond faster and more innovatively for better decision making (Cortellazzo et al., 2019). Darics (2020) highlighted that in a remote work environment, management and leadership functions are combined and managers must manage performance and implement solutions when needed and create and maintain a team identity by establishing and sharing a vision, corporate values, and organizational goals into a trusting working environment. Moreover, in teleworking, considering a reduction in the social and interpersonal distance, leaders should be more democratic with access to information and willing to keep an open communication (Montgomery et al., 2016). In this context, the adaptive structuration theory (DeSanctis and Poole, 1994) suggests that many organizational phenomena including organizational leadership transform when interacting with Advanced Information Technologies (AITs). From this approach, AITs mediate leadership influence and create an integrated mechanism of leadership and management. In fact, from a management perspective, AITs can have various purposes, including sharing information, planning, record keeping, or data analysis. From a leadership perspective, effective leaders at e-leadership positions are successful when they can use various AITs to achieve greater performance, enhance employees’ job satisfaction while reducing the rates of turnover (Montgomery et al., 2016).

## E-Leadership and Its Conceptualization

Electronic or e-leadership is not just an extension of traditional leadership but also implies a crucial change in how leaders and followers relate to each other within the organizations and with stakeholders (Avolio and Kahai, 2003), making it imperative for leaders to change their practices (Malhotra et al., 2007). Kahai et al. (2013) asserted that scholars should go beyond traditional leadership theories to explain the role of leaders and leadership in remote work environments. E-leadership implies the development of distinct abilities to improve organizational functioning in virtual work environments (Roman et al., 2019). For e-leaders, the known social skills, such as the characteristics of effective face-to-face communication may not be enough to lead in virtual environments, where these characteristics must be complemented with the skills to manage various virtual communications platforms. However, Liu et al. (2020) asserted that many propositions used in generic leadership theories can be applied to e-leadership. This premise should be tested to build a genuine theory of e-leadership. Dulebohn and Hoch (2017) highlighted the need for developing a new theory and conducting empirical research to help organizations in designing, structuring, and managing virtual teams.

Cortellazzo et al. (2019) state that there is no shared approach to study and theorize about this phenomenon. However, because e-leadership is a multidimensional phenomenon, it should be studied from different disciplines, avoiding fragmented knowledge, and from different levels of analysis: macro (e-leadership and organization) and micro (e-leader’s skills and leading virtual teams). Thus, as asserted by Liu et al. (2020), e-leadership is an important trend not only for the rapid progress in technology and its application during the pandemic but also presents a challenge for companies to adopt the technology, that is, to benefit from its advantages. These authors stated that if this process is not well addressed by leaders and used only to impose mandates, e-leadership could increase alienation and chaos. Up to now, hybrid teleworking (work from home few days a week) appears to provide the best balance between remote work flexibility and benefits of working face to face with management and coworkers. However, more evidence is needed (Bentley, 2014). Supporting this view, a study conducted in Australia on teleworking, productivity results showed that employees preferred a maximum of 1–3 days away from the office as the most feasible telework arrangement (Bosua et al., 2017).

Some years ago, e-leadership was described as an ineludible challenge for companies (Esguerra and Contreras, 2016). The “quiet revolution,” as named by Avolio and Kahai (2003), occurred to companies much earlier. Being prepared for virtual work environments was a priority to respond to a globalized world immersed in the digital era. Now during the pandemic and onward, it is crucial for business survival. Thus, e-leadership will be a relevant challenge that companies must face for success and sustainability. E-leadership is an irreversible trend that is here to stay.

Leadership as a field of study has largely focused on organizations where employees are working on site. Studies on leadership and teleworkers are scarce. Avolio et al. (2014) stated that the study of e-leadership is in the early stage of development. Van Wart et al. (2019) asserted that the study on how the current digital revolution is changing the relationship between leaders and followers has been modest. Interestingly, though, from 2001 to date, there are 102 published articles related to e-leadership in the Web of Science Core Collection. Of these, only 32 papers included the term e-leadership in their title. In their seminal work, Avolio et al. (2000) defined e-leadership “as a social influence process mediated by AIT to produce a change in attitudes, feelings, thinking, behavior, and/or performance with individuals, groups, and/or organizations” (p.617). Similarly, Al-jedaibi (2001) explained e-leadership as the kind of leadership in the e-environment context where work is mediated by information technologies, especially the Internet. However, the leader is not necessarily a “tech guru.” He or she only should know how to benefit from high technology and lead efficiently through technology. Gurr (2004) also focused on e-leadership and claimed that technology-mediated environments require unique leaders who are good at coping with complexity. They should establish a suitable social climate with sustained communication and can demonstrate exemplary interpersonal skills through related technology. Recently, Cortellazzo et al. (2019) stated that in spite of the advances, there is no well-established and consensual definition of e-leadership.

Cowan (2014) proposed that effective e-leadership should be characterized by building trust with each member of the team and establishing a virtual “presence” preventing distance from becoming a barrier. Similarly, e-leaders should address the teams’ social-emotional needs and their members and promote healthy teams through interactions. E-leaders should develop effective communication skills, that is, select a suitable communication tool, provide relevant and contextual communication considering possible cultural differences, provide positive feedback to the teams, and recognize their performance. Nayani et al. (2018) asserted that besides high levels of instrumental support and competent communication, leaders should promote trust using motivational language. More recently, Roman et al. (2019) asserted that effective e-leaders should communicate clearly, promote adequate social interactions, know how to use the technological media, be able to build responsible teams, inspire change, and develop trust virtually. Van Wart et al. (2019) defined e-leadership as “…the effective use and blending electronic and traditional methods of communication. It implies an awareness of current ICTs, selective adoption of new ICTs for oneself and the organization, and technical competence in using those ICTs selected.” (p.83). According to the authors, effective e-leadership is not only use of ICTs but also implies that when this media offers the best advantages, select the most appropriate one, based on the needs, using face-to-face communication channels where more appropriate, integrating distance and non-distance methods, according to the purposes.

Van Wart et al. (2019) conceptualized e-leadership as the effective use and blending of electronic and traditional methods of communication and proposed the definition of e-leadership through the following competencies that should be empirically tested: (1) Communication skills (communication clarity, avoidance of miscommunication, management of communication flow), (2) Social skills (leaders’ support), (3) Team building skills (encompassing team motivation, team accountability, and team member recognition), (4) Change management skill (covering change techniques), (5) Technological skills (correct use of relevant ICTs, blending traditional and virtual methods, technological knowledge, and technological security) and (6) Trustworthiness (sense of trust, honesty, consistency, follow-through, fairness, integrity, work-life balance, and support of diversity).

In virtual or remote work environments, leaders should demonstrate a more inclusive leadership style (Schwarzmüller et al., 2018). For e-leaders, the social skills, such as the characteristics of effective face-to-face communication, may not suffice to lead in virtual environments (Roman et al., 2019). Cortellazzo et al. (2019) highlighted that e-leaders should develop a communication where employees feel free to present their ideas, allowing them to participate in the decision-making process and encourage autonomy, collaboration, and responsibility, and promoting a positive organizational environment with their leadership. In this new work environment, information is more visible and easier to share, allowing employees to be more independent in their work. Thus, companies not only benefit from employees’ good performance but reduce the need to supervise them (Schwarzmüller et al., 2018).

In this regard, Roman et al. (2019) defined e-communication as the ability to communicate properly through ICTs, avoiding errors or excesses that affect good performance. This ability is marked by the use of an appropriate tone, providing clear messages to employees through the right communication media. These authors also suggested that this process involves technical issues, such as selecting the best method to communicate considering the richness of the tool, the receiver’s preferences, and decide upon the use of synchronous or asynchronous methods. With regard to the use of synchronous or asynchronous methods, both temporary forms of communication offer advantages. For example, asynchronous communication allows a continuous flow of information (Gupta and Pathak, 2018). Additionally, Cortellazzo et al. (2019) highlighted the importance of maintaining clear norms of communication, having regular interaction with the teams, providing positive feedback, avoiding ambiguous messages, and conducting good supervision of each member’s contribution. In contrast, deficient communication from leaders may lead to unknown situations, leaving employees with a feeling of helplessness (Wojcak et al., 2016). The e-social environment is the second important property of e-leadership (Roman et al., 2019), that is, creating a positive work atmosphere with a sense of connectedness with the group to increase communication and collaboration through digital communication methods. Through e-social characteristics of e-leadership, isolation among team members can be successfully prevented (Walther and Bazarova, 2008). Furthermore, the e-change property refers to the e-leaders’ capability of making noteworthy changes required for adaptation of AITs. While the e-team property of e-leadership is about a leader’s capabilities in creating accountable, satisfied, and efficient teams in virtual business environments, e-technological skills are also important e-leadership properties. It is the competency of an e-leader to be aware of novel technologies, being able to keep up with relevant technological developments, and embracing high-level cyber security (Roman et al., 2019).

Finally, another important characteristic of e-leadership is the capacity to innovate. E-leaders should be able to identify the need for change and promote innovation in their organizations and teams (Schwarzmüller et al., 2018). However, e-leaders must be careful that these continuous changes do not disrupt the company’s focus and its mission. Therefore, these leaders should be flexible, innovative, have clarity about the organization’s goals (Cortellazzo et al., 2019). Table 2 presents the main issues related to e-leadership.

**TABLE 2:** Main issues about e-leadership.

Main issues
E-leadership is not an extension of traditional leadership (Avolio and Kahai, 2003).
It is a priority to build and share a genuine theory of e-leadership (Dulebohn and Hoch, 2017).
There is no well-established and consensual definition of e-leadership (Cortellazzo et al., 2019).
E-leadership has to be studied from different disciplines (Cortellazzo et al., 2019).
Studies on e-Leadership are still scarce, the knowledge of this topic is in an early stage of development (Avolio et al., 2014; Van Wart et al., 2019).
Some characteristics of generic leadership theories could be applied to e-leadership (Liu et al., 2020).

## E-Leadership, Teleworking and Virtual Teams

As mentioned before, teleworking is a new form of work organization that gained ground in most organizations around the world due to the pandemic, increasing distance in the interpersonal relations in the work environment. This way of working offers huge opportunities to companies, but a huge challenge to leaders who have to lead an environment of boundaryless work through technology. This challenge implies that both leaders and followers develop technical competencies to facilitate the monitoring, coordination, and alignment of work through novel technology-supported structures, in order to diminish barriers (Alfehaid and Mohamed, 2019). For this purpose, e-leaders have to be competent with the latest ICTs (Groysberg, 2014) E-leaders not only have the responsibility to adopt internet-based computer technologies in their organizations but also have to create awareness regarding these technologies to make teleworking possible and convenient (Van Wart et al., 2019).

To take advantage of the possibilities that teleworking offers, companies cannot be led in the same way as has been done traditionally. De Vries et al. (2019) indicates that hierarchical forms of leadership are less suitable in virtual work environments. Traditional leadership is supported in social influence mechanisms. However, in virtual environments this influence is mediated by computer technologies producing changes in behaviors, emotions, thoughts, and performance of workers (Van Wart et al., 2019). In remote work settings, e-leaders cannot be oriented to organize fragmented tasks; they have to be close to their employees reducing the negative impact that produces the physical and psychological distances (Stokols et al., 2009). Similarly, Maciel et al. (2017) stated that effective e-leadership encourages the performance in teleworking by minimizing the distance between the organization and its employees and brings the organization and its customers closer with the help of high technology. To reach that, e-leaders have to develop trust in their relationships, allowing greater exchange of ideas; they encourage information flow, and generate creative solutions (Avolio et al., 2014). Likewise, findings of Panteli et al. (2019) showed that e-leaders boost employees’ work engagement through effective use of resources and their attitude of development, support, and nourishment. These properties are helpful in contexts characterized by greater geographic distance, diversity, some ambiguity, and unfamiliarity with remote working. Moreover, through the delegation and the effective provision of feedback, e-leaders develop and support their spatially dispersed and sometimes, socially distanced employees. As Kahai et al. (2013) suggest e-leaders with their behaviors can relieve the potential problems of teleworking such as the greater physical and social distance that makes social interactions difficult. Even though in the related literature most of the researchers are focused on the importance of e-leaders to provide emotional and technological support to their employees (Friedman and Westring, 2015; Bentley et al., 2016), some noteworthy studies are focused on the need to provide ergonomics support to the employee’s home office which, in turn, has been related to talent retention of teleworkers (Eversole et al., 2012; Allen et al., 2015).

Virtual team is an attendant concept of e-leadership (DasGupta, 2011). An important challenge for e-leaders is to build effective, autonomous, interdependent (Cortellazzo et al., 2019), and committed virtual teams (Politis, 2014) for which trust is crucial. Virtual teams include members who are geographically dispersed but working together in an interdependent task through electronic means with low face-to-face interaction (Malhotra et al., 2007). Diverse virtual teams have the challenge of coordinating tasks across different locations, time zones, and cultures (Siebdrat et al., 2014). In fact, managing a distributed workforce creates heightened leadership challenges (Hoegl and Muethel, 2016). The inclusion of digital media in the companies, affects their design of work and the way employees work together in effective virtual teams (Schwarzmüller et al., 2018). Because of the pandemic, e-leadership is required more than face-to-face leadership. However, in the future, virtual teams would persist due to the opportunities they offer. Regardless of the leadership style, similar to in person, leaders of virtual teams should articulate and communicate the vision with passion, shaping a culture based on organizational values; however, the method is still unclear. Even in developed countries, there is a lack of knowledge of e-leadership skills needed to address successful virtual teams in complex work processes (Liu et al., 2020). Thus, how e-leaders can build effective virtual teams is a relevant challenge to the leadership field.

Leading virtual teams effectively offer enormous competitive advantages for the companies. The possibility of building effective teams consisting of people with different experiences, from diverse cultures and knowledge of different fields, regardless of the time and distance, is enormous. Nayani et al. (2018) explained that although distributed workers are diverse, they share common work characteristics of temporospatial distance from coworkers, managers, and leaders. A virtual environment provides opportunities to interact and establish connections with people around the world (Cortellazzo et al., 2019). Malhotra et al. (2007) claimed that this possibility allows thinking globally and acting locally, showing the creative capacity of such a virtual team. However, because the national culture impacts leadership (Dorfman and House, 2004), the geographical dispersion and cultural diversity between team members can be a barrier to building trust within the teams (Gupta and Pathak, 2018). Indeed, the physical distance and cultural diversity threaten trust building among the team members, affecting their commitment and cohesion (Hoch and Kozlowski, 2014). In this regard, e-leaders should develop intercultural competences to communicate adequately with team members and build trust through interrelationship. A virtual team leader should develop cross-cultural skills to understand different cultures, their similarities, and differences (Schwarzmüller et al., 2018). Nevertheless, there is a need for further research on the impact of culture on e-leadership (Cowan, 2014). Under effective e-leadership, such diversity in the teams increases the members’ innovative behavior and will influence the companies’ innovation. In this regard, more than traditional leaders, e-leaders should lead diversity if they must leverage the advantage offered by virtual teams. In this regard, Gupta and Pathak (2018) asserted that team members’ heterogeneity promotes creativity and innovation through a combination of various perspectives to achieve an objective. Another important challenge for e-leaders is to recruit, retain, reward, and motivate globally talented employees to maintain their competitive advantage in the globalized world (Avolio et al., 2014).

Similar to traditional teams, leading a virtual team requires leadership and management skills. As Nayani et al. (2018) asserted, organizations should ensure occupational safety and health of teleworkers through appropriate management (i.e., systems, procedures, and practices) and effective leadership practices. However, there is a paucity of research in this field and its results are fragmented. Leading virtual teams has an additional challenge because leaders should ensure that each team member is committed to the project and gives the best according to his or her expertise (Malhotra et al., 2007). Recently, Schwarzmüller et al. (2018) highlighted that e-leaders should develop tolerance to the ambiguity and be creative in establishing the organizational structures and processes that assure that all members of virtual teams are working for the shared objective. Supporting this view, Darics (2020) claimed that e-leaders have two important roles: (1) managing performance and implementing novel solutions to work-related problems, and (2) creating and maintaining group identity by establishing a shared mission, vision, values, and goals. Thus, Malhotra et al. (2007) proposed six leadership practices to have successful virtual teams: (1) establish and maintain the thrust through technology; (2) appreciate and understand the diversity; (3) manage the work-life cycle well through meetings; (4) monitor progress of teamwork; (5) enhance the visibility of the team members (within and outside of the team), and (6) allow individual members to avail of the benefits from the teamwork.

Jones and O’shea (2004) stated that the hierarchical leadership approaches in e-teams have limitations in terms of providing flexibility to group members during the process of collaboration. In virtual environments, e-leaders should distribute the leadership well within the teams. This allows teams to shape their own leadership style and promote the collective development of leadership (Gupta and Pathak, 2018). However, sharing leadership does not exclude the formal leader figure but assumes that any member can lead the team, follow up, and make the best decision for the team (Cortellazzo et al., 2019). Through shared leadership, not just the team leader but also team members take responsibility and assume authority to consider both their own spheres of work and the entire project (Hoegl and Muethel, 2016). Shared leadership promotes team members’ identification within the group and initiates action flows for goal achievement. However, for shared leadership, the leader should realize and appreciate members’ potential and willingness to assume the responsibility of a few leadership duties (Hoegl and Muethel, 2016). Finally, communication in virtual teams is more complex than in traditional teams that use face-to-face communication. In most virtual teams, e-leaders should communicate and work asynchronously through AITs. Hence, time and space separation in virtual teams create important challenges for leaders by demanding extra leadership competencies in ensuring and promoting organizational management (Fan et al., 2014). Given that the coordination of virtual teams for task accomplishment, responsibility, and knowledge sharing is done through telecommunication technologies, sometimes there may be distortion in information interpretation leading to misunderstandings and employee demotivation. Thus, e-leaders should be highly competent in their verbal communications to motivate their employees (Fan et al., 2014). Virtual team leaders should avoid employees’ feeling of isolation and promote team cohesion. This implies adequate establishment of norms of collaboration, knowledge sharing, recognition, and rewarding the teams and their members (Malhotra et al., 2007) to be “present” socially and emotionally (Cowan, 2014).

## Conclusion and Propositions for Further Studies

The pandemic has increased the need to augment our knowledge on how to lead effectively and build highly functional virtual teams. Despite being recognized much earlier, there is limited knowledge on e-leadership and no theory specific to such leadership. It is unclear whether the current knowledge on leadership can be applied to e-leadership. Similarly, results from various studies on the effectiveness of e-leadership and its effects on employees have been inconclusive. There is some consensus that leaders should consider giving the opportunity to some employees to telework when the job or the task can be done out of the workplace and to avail of the benefits of this mode of work. Thus, as a result of applied research, it is imperative to create profiles of eligibility to telework. In other words, people who can leverage the advantage of working remotely must establish different levels of attendance based on the work or task (e.g., once a week, some days a week, or full-time).

The revised literature highlighted the importance of achieving a better understanding of the effects of teleworking on employees’ well-being and organizational performance. Currently, due to the pandemic, there is a huge global interest in studying this topic from the perspective of both practitioners and researchers. It is needed to conduct studies that rigorously examined teleworking and e-leadership and the reasons for success and even for the failures to learn more about how to manage this new way to work. However, there is a paucity of knowledge on the outcomes of such a method of work, and its results have been inconclusive. For example, Narayanan et al. (2017) mentioned that companies such as Hewlett-Packard, Yahoo, and Best Buy reduced the hours of teleworking and asked workers to return to the traditional workplace. Case studies are needed to understand these failed experiences.

Finally, one of the main weaknesses in the studies of teleworking and e-leadership is their methodology, small samples are not representative, and robust theoretical foundations are scarce. It is important to improve methodological rigor for acquiring reliable and valid data. More than descriptive or correlational studies are necessary. More experimental and quasi-experimental studies are needed as well as more longitudinal studies and mixed methods for better comprehension of the phenomena.

Due to the availability of a global workforce, it is important to conduct cross-cultural studies and analyze the role of e-leadership and cultural differences. As Narayanan et al. (2017) suggested, research should be conducted on psychology and sociology and topics such as social isolation, group, and team behavior and management practices in teleworking. How to promote trust through organizational culture and leadership should be examined. At the individual level, research on psychology should be conducted to understand the personality, qualities, skills, and cognitive needs of those employees who are more suitable to work remotely and conduct financial research for the cost-benefit analysis of teleworking. Liu et al. (2020) and Cortellazzo et al. (2019) suggested that a theory of e-leadership, sharing approaches, and theorizing about this phenomenon is needed. Finally, based on Nayani et al. (2018), the teleworkers’ health is another promising line of research that should be developed through robust conceptual frameworks and rigorous methods.

In sum, from the theoretical perspective, further studies should help to build a theory of e-leadership that is common for all researchers in this topic. In this way, findings around the world can be contrasted, which will contribute to building a solid body of knowledge of how to lead in virtual environments. These studies will help also e-leaders to develop their intercultural competencies to lead in global environments. Likewise, the methodology of empirical studies should be strengthened to conduct research in controlled settings (research in a laboratory) making relevant contributions that explain how to successfully lead in virtual environments. In fact, the body of knowledge that will continue to be built in the next years will allow to identify and test the competencies that need to be developed by e-leaders in order to be effective as leaders and efficient as managers in this new way of work, which apparently will be kept to varying degrees once the pandemic is overcome. As a result of these studies, leaders can be trained and human resource managers can be guided in order to increase organizational performance while improving the employees’ well-being in a healthy work environment.

## Author Contributions

FC was engaged in the investigation, literature search and selection, writing original draft, preparation, and finishing the last version. EB was involved in investigation, literature search and selection, contribution to the original draft, and contribution to the last version. GA was engaged in investigation, literature search and selection, contribution to the original draft, and contribution to the last version. All authors contributed to the article and approved the submitted version.

## Conflict of Interest

The authors declare that the research was conducted in the absence of any commercial or financial relationships that could be construed as a potential conflict of interest.
